# Dimethyl itaconate, an itaconate derivative, exhibits immunomodulatory effects on neuroinflammation in experimental autoimmune encephalomyelitis

**DOI:** 10.1186/s12974-020-01768-7

**Published:** 2020-04-29

**Authors:** Ping-Chang Kuo, Wen-Tsan Weng, Barbara A. Scofield, Hallel C. Paraiso, Dennis A. Brown, Pei-Yu Wang, I-Chen Yu, Jui-Hung Yen

**Affiliations:** 1 Department of Microbiology and Immunology, Indiana University School of Medicine, 2101 E. Coliseum Boulevard, Fort Wayne, IN 46805 USA; 2 Department of Anatomy, Cell Biology and Physiology, Indiana University School of Medicine, Fort Wayne, IN USA; 3 Department of Pharmaceutical Sciences, Manchester University College of Pharmacy, Natural and Health Sciences, Fort Wayne, IN USA; 4 Graduate Institute of Brain and Mind Science, College of Medicine, National Taiwan University, Taipei, Taiwan

**Keywords:** Itaconate, DMI, MS/EAE, Microglia, Th1/Th17, Neuroinflammation, Blood-brain barrier

## Abstract

**Background:**

Inflammatory stimuli induce immunoresponsive gene 1 (IRG1) expression that in turn catalyzes the production of itaconate from the tricarboxylic acid cycle. Itaconate has recently emerged as a regulator of immune cell functions, especially in macrophages. Studies show that itaconate is required for the activation of anti-inflammatory transcription factor Nrf2 by LPS in mouse and human macrophages, and LPS-activated *IRG1*^*-/-*^ macrophages that lack endogenous itaconate production exhibit augmented inflammatory responses. Moreover, dimethyl itaconate (DMI), an itaconate derivative, inhibits IL-17-induced IκBς activation in keratinocytes and modulates IL-17-IκBς pathway-mediated skin inflammation in an animal model of psoriasis. Currently, the effect of itaconate on regulating macrophage functions and peripheral inflammatory immune responses is well established. However, its effect on microglia (MG) and CNS inflammatory immune responses remains unexplored. Thus, we investigated whether itaconate possesses an immunomodulatory effect on regulating MG activation and CNS inflammation in animal models of multiple sclerosis, experimental autoimmune encephalomyelitis (EAE).

**Methods:**

Chronic C57BL/6 EAE was induced followed by DMI treatment. The effect of DMI on disease severity, blood-brain barrier (BBB) disruption, MG activation, peripheral Th1/Th17 differentiation, and the CNS infiltration of Th1/Th17 cells in EAE was determined. Primary MG was cultured to study the effect of DMI on MG activation. Relapsing-remitting SJL/J EAE was induced to assess the therapeutic effect of DMI.

**Results:**

Our results show DMI ameliorated disease severity in the chronic C57BL/6 EAE model. Further analysis of the cellular and molecular mechanisms revealed that DMI mitigated BBB disruption, inhibited MMP3/MMP9 production, suppressed microglia activation, inhibited peripheral Th1/Th17 differentiation, and repressed the CNS infiltration of Th1 and Th17 cells. Strikingly, DMI also exhibited a therapeutic effect on alleviating severity of relapse in the relapsing-remitting SJL/J EAE model.

**Conclusions:**

We demonstrate that DMI suppresses neuroinflammation and ameliorates disease severity in EAE through multiple cellular and molecular mechanisms, suggesting that DMI can be developed as a novel therapeutic agent for the treatment of MS/EAE through its immunomodulatory and anti-inflammatory properties.

## Background

Multiple sclerosis (MS) is an autoimmune disorder characterized by immune-mediated demyelination triggered by the CNS infiltrating myelin-specific pathogenic T cells and subsequent neuroinflammation [1–3]. Experimental autoimmune encephalomyelitis (EAE) is a commonly used animal model to study MS. Studies have demonstrated that several T cell subsets, including CD4^+^ T cell lineages of Th1 and Th17, CD8^+^ T cells, and IL-17-producting γδT cells, contributed to the immunopathogenisis of EAE [4–8]. In addition, antigen presenting cells, such as dendritic cells, macrophages, and microglia (MG), have been shown to play a pivotal role in the induction of encephalitogenic T cells through releasing essential inflammatory cytokines, such as IL-12 and IL-23 for Th1 and Th17 differentiation, respectively [9].

Itaconate is a metabolite produced by immune cells especially macrophages upon activation. It has been shown that inflammatory stimuli induce immunoresponsive gene 1 (IRG1) expression that in turn catalyzes the production of itaconate from the tricarboxylic acid cycle. Itaconate has recently emerged as a regulator of macrophage function. For instance, it has been shown that itaconate is required for the activation of anti-inflammatory transcription factor Nrf2 by LPS in mouse and human macrophages [10]. In addition, LPS-activated *IRG1*^*-/-*^ macrophages that lack endogenous itaconate production exhibit augmented inflammatory response when compared to LPS-activated wild-type macrophages [11]. Moreover, a recent study demonstrates that in addition to its regulatory effects on immune responses through activating Nrf2/HO-1 pathway, itaconate is capable of modulating immune responses through an Nrf2-independent mechanism. The study shows that dimethyl itaconate (DMI), a cell-permeable itaconate derivative, inhibits IL-17-induced IκBς activation in keratinocytes, and the immunomodulatory effect of DMI on regulating the IL-17-IκBς axis-induced inflammation can also be observed in the imiquimod-induced psoriasis animal model [12].

MG make up the innate immune system of the CNS and are the key cellular mediators of neuroinflammatory processes [13–15]. MG activation leads to increased secretion of inflammatory cytokines and enhanced expression of surface maturation markers. In MS, homeostatic MG were completely lost in active and slowly expanding lesions. In contrast, activated MG with elevated expression of pro-inflammatory genes were highly observed around lesions in the brain of MS patients [16], suggesting that MG activation is linked to the disease development of MS.

Currently, the effect of itaconate on regulating macrophage functions and peripheral inflammatory immune responses is well established. However, its effect on MG and CNS inflammatory immune responses remains unexplored. In the present study, we investigated whether itaconate possesses an immunomodulatory effect on regulating MG activation and CNS inflammation in EAE. We reported for the first time that DMI, a derivative of itaconate, not only ameliorated disease severity in chronic EAE but also mitigated disease relapse in relapsing-remitting EAE. In addition, we identified that DMI suppressed MG activation, attenuated neuroinflammation, lessened BBB disruption, inhibited peripheral Th1/Th17 differentiation, and repressed the CNS infiltration of Th1/Th17 in EAE.

## Methods and materials

### Animals

C57BL/6 and SJL/J mice used in this study were purchased from The Jackson Laboratory (Bar Harbor, ME). Mice were housed in animal housing rooms with controlled temperature, humidity, and 12 h∶12 h light∶dark cycle and bred with free accessed food and water. All animal-associated studies were approved by the Purdue University Animal Care and Use Committee.

### Reagents

Dimethyl itaconate (DMI), Complete Freund’s adjuvant, Evans blue, trichloroacetic acid (TCA), phorbol myristate acetate (PMA), ionomycin, and paraformaldehyde were purchased from Sigma-Aldrich (St. Louis, MO). Antibodies of Alexa Fluor 488 anti-mouse CD4 (clone: RM4-5), APC anti-mouse IL-17A (clone: TC11-18H10.1), PE/Cy7 anti-mouse IFNγ (clone: XMG1.2), APC anti-mouse CD45 (clone: 30-F11), PE anti-mouse CD11b (clone: M1/70), PE/Cy7 anti-mouse CD80 (clone: 16-10A1), PE/Cy7 anti-mouse CD86 (clone: GL-1), PE anti-mouse CD25 (clone: PC61), Alexa Fluor 647 anti-mouse Foxp3 (clone: MF-14), anti-mouse CD68 (clone: FA-11), anti-mouse MMP9 (clone: L51/82), anti-mouse MMP3 (clone: M4405F10), anti-mouse CD3 (clone: 145-211), anti-mouse CD28 (clone: 37.51) and anti-mouse IFNγ (clone: R4-6A2), reagents of brefeldin A solution, fixation buffer and permeabilization wash buffer, and recombinant cytokines of mouse IL-12, mouse IL-6, and human TGFβ were purchased from BioLegend (San Diego, CA). Anti-IBA1 antibody was purchased from Fujifilm Wako Chemicals (Richmond, VA). Alexa Fluor 488- and Alexa Fluor 647-conjugated secondary antibodies and ProLong Gold antifade mountant containing DAPI were purchased from Invitrogen, Thermo Fisher Scientific (Waltham, MA, USA). Antibodies of anti-mouse Nrf2, anti-mouse HO-1, and anti-mouse GAPDH were purchased from Proteintech (Rosemont, IL). *Mycobacterium tuberculosis* H37 RA and anti-mouse β-actin antibody were purchased from BD Biosciences (San Jose, CA). Pertussis toxin was purchased from List Biological Labs (Campbell, CA). Percoll was purchased from GE Healthcare Life Sciences (Pittsburgh, PA).

### EAE induction

For chronic EAE induction, female C57BL/6 mice with an age of 8–9 weeks old were injected subcutaneously with an emulsion of 200 μg MOG_35–55_ peptide in Complete Freund’s adjuvant containing *Mycobacterium tuberculosis* H37 RA (final concentration 2 mg/ml) on day 0 followed by intraperitoneal administration with 200 ng pertussis toxin on day 0 and day 2. For relapsing-remitting EAE induction, female SJL/J mice with an age of 8–9 weeks old were injected subcutaneously with 100 μg PLP_139–151_ peptide in Complete Freund’s adjuvant containing *Mycobacterium tuberculosis* H37 RA (final concentration 2 mg/ml) on day 0 followed by intraperitoneal administration with 200 ng pertussis toxin on day 0 and day 2. The immunized mice were randomly grouped and administered intraperitoneally (i.p.) with vehicle (DMSO) or DMI every day, starting from day 3 post-immunization (C57BL/6 model) or starting from the first remission when animals reached the disease score of ≤ 1.5 (SJL/J model). C57BL/6 and SJL/J EAE mice were subjected to clinical score evaluation everyday based on the following criteria: 0: normal mouse and no overt signs of disease, 1: limp tail or hind limb weakness, 2: limp tail and hind limb weakness, 3: partial hind limb paralysis, 4: complete hind limb paralysis, 5: moribund state. Animals with clinical scores of 5 were euthanized.

### Evans blue BBB permeability assay

The Evans blue permeability assay was conducted as previously described [17]. EAE mice were administered intravenously (i.v.) with 4 ml/kg 2% (w/v) Evans blue dye/0.9% saline solution from lateral tail vein at day 12 post-immunization. Two hours after Evans blue injection, mice were anesthetized and perfused with PBS buffer. The brain and spinal cord were harvested and subjected to imaging to observe Evans blue leakage. The spinal cord tissues were then weighted and homogenized in 50% TCA solution followed by incubation at 4 °C for overnight. Following centrifugation, the supernatants were collected and diluted with 95% ethanol in the ratio of 1:3. The amount of Evans blue in the spinal cord tissues was determined by measuring the fluorescence with excitation at 540/25 nm and emission at 645/40 nm (BioTek Synergy HT microplate reader).

### Cell cultures

Primary MG were obtained following procedures described previously [18]. Briefly, cerebral cortical cells were collected from 1 to 2 days old neonatal mice and seeded in T75 flasks containing Dulbecco Modified Eagle Medium/F12 (HyClone™) with 2 mM glutamine, 1× antibiotic/antimycotic, and 10% heat-inactivated FBS (complete medium). After removing medium, cells were replenished with fresh complete medium containing 10 ng/ml GM-CSF on day 3 and 6 after plating. MG were collected by shaking the flasks at 250 rpm for 30 min at 37 °C on day 13 or 14. After seeding, MG were treated according to the conditions described. BV2 mouse brain microglial cell lines obtained from American Type Culture Collection were grown to confluence. After trypsinization, cells were then seeded onto tissue culture plates followed by stimulation as described.

### Western blot analysis

Cells or tissue samples were lysed in radioimmunoprecipitation assay buffer [50 mM Tris-HCl (pH 8.0), 150 mM NaCl, 1% NP-40, 0.5% sodium deoxycholate] plus 1X protease inhibitor cocktail III (Alfa aesar) with 0.1% SDS (cells) or 0.3% SDS (tissues). Protein concentrations were measured by using Pierce™ BCA Protein Assay Kit (Thermo Fisher Scientific). Ten micrograms (cells) or 50 μg (tissues) of whole protein lysate was mixed with sample buffer, boiled for 10 min, and loaded on 10% SDS-PAGE gels followed by electrophoresis. Separated proteins were transferred to polyvinylidene difluoride membranes (Millipore) and then probed with antibodies against MMP9, MMP3, GAPDH, Nrf2, HO-1, or β-actin in blocking buffer. HRP-conjugated goat anti-rabbit IgG or anti-mouse IgG antibodies (BD Biosciences) were used as secondary antibodies. The membranes were then incubated with Immobilon™ Western Chemiluminescent HRP Substrate (Millipore) followed by the detection of protein signals by using X-ray films. The quantification of protein signal was measured by using ImageJ.

### Immunohistochemistry

Paraffin-embedded lumbar regions of spinal cord samples were cut into 8 μm sections by the microtome (Leica RM2155). The slides were deparaffinized followed by antigen retrieval with 10 mM citrate buffer for 20 min at 90 °C. The slides were then incubated with anti-IBA1 antibody (1:500) at 4 °C overnight. After incubation, the sections were rinsed with PBS and incubated with Alexa 488-conjugated secondary antibody (1:1000) for 1 h at room temperature (RT). After washing with PBS, samples were coverslipped with ProLong Gold antifade mountant containing DAPI. Two sections from each spinal cord sample were stained. Immunofluorescence images were captured with fluorescence microscope (BX53, Olympus; camera: EXi Aqua, Q Imaging), and the number of IBA1^+^ cells per mm^2^ was then counted and quantified by imageJ.

### Immunocytochemistry

MG were fixed with 2% paraformaldehyde for 15 min at RT and then permeabilized with 0.25% Triton X-100 for 10 min. After blocking with 5% normal donkey serum for 30 min at RT, MG were incubated with anti-mouse CD68 (1:500) or anti-mouse HO-1 (1:50) for 2 h at RT. MG were then rinsed with PBS and incubated with Alexa Fluor 488- or Alexa Fluor 647-conjugated secondary antibody (1:1000) for 1 h. After washing with PBS, samples were coverslipped with ProLong Gold antifade mountant containing DAPI. Immunofluorescence images were then captured with the Fluoview FV10i confocal microscope (Olympus).

### Real-time quantitative PCR (Q-PCR)

Primary MG were subjected to RNA extraction followed by cDNA synthesis. The mRNA expression of *Il-1α*, *Il-1β*, *Il-12p35*, *Il-12p40*, *Il-23p19*, and *Gm-csf* was measured by Q-PCR. The primers used were *Il-1α*: sense 5′-CGCTTGAGTCGGCAAAGAAAT-3′ and antisense 5′-CTTCCCGTTGCTTGACGTTG-3′; *Il-1β*: sense 5′-CCCTGCAGCTGGAGAGTGTGGA-3′ and antisense 5′-TGTGCTCTGCTTGGAGGTGCTG-3′; *Il-12p35*: sense 5′-CTGTGCCTTGGTAGCATCTATG-3′ and anti-sense 5′-GCAGAGTCTCGCCATTATGATTC-3′; *Il-12p40*: sense 5′-TGGTTTGCCATCGTTTTGCTG-3′ and antisense 5′-ACAGGTGAGGTTCACTGTTTCT-3′; *Il-23p19*: sense 5′-TGCTGGATTGCAGAGCAGTAA-3′ and anti-sense 5′-GCATGCAGAG ATTCCGAGAGA-3′; *Gm-csf*: sense 5′-ATGCCTGTCACGTTGAATGAAG-3′ and anti-sense 5′-GCGG GTCTGCACACATGTTA-3′.

### Isolation of mononuclear cells from brain and spinal cord tissues

Mononuclear cell isolation was processed as previously described [19]. Briefly, at day 12–14 post-immunization, vehicle- and DMI-treated EAE mice were anesthetized, and the brain and spinal cord were then harvested and homogenized with 1× HBSS buffer followed by filtration through 70 μm nylon cell strainers. After centrifugation, cells were resuspended in 30% Percoll and underlayered with 70% Percoll. The samples were then centrifuged at RT for 25 min at 1000 g, and the mononuclear cells were isolated from the interface between 30% and 70% Percoll followed by FACS analysis.

### In vitro Th1 and Th17 differentiation

Splenocytes (2 × 10^6^ cells/well) isolated from the spleen of C57BL/6 mice were activated with plate-coat anti-mouse CD3 antibody (3 μg/ml) and soluble ant-mouse CD28 antibody (2 μg/ml) in the presence of IL-12 (10 ng/ml) for Th1 differentiation or IL-6 (20 ng/ml), TGFβ (10 ng/ml), and anti-mouse IFNγ antibody (5 μg/ml) for Th17 differentiation. Forty-eight hours later, cells were then collected and subjected to FACS analysis for intracellular expression of IFNγ and IL-17 in CD4^+^ cells.

### FACS analysis for surface markers, intracellular cytokines and Foxp3 expression

For MG surface marker analysis, isolated mononuclear cells were stained with PE anti-mouse CD11b and APC anti-mouse CD45 in the combination with PE/Cy7 anti-mouse CD80 or PE/Cy7 anti-mouse CD86 followed by FACS analysis. For intracellular cytokine analysis, cells isolated from spleens and cervical lymph nodes or mononuclear cells isolated from the spinal cord and brain of C57BL/6 EAE animals were stimulated with PMA (50 ng/ml), ionomycin (750 ng/ml), and brefeldin A solution (1 μl/ml). After 5 h of stimulation, cells were then fixed and permeabilized followed by staining with antibodies of Alexa Fluor 488 anti-mouse CD4, PE/Cy7 anti-mouse IFNγ, and APC anti-mouse IL-17. The intracellular expression of IFNγ and IL-17 in CD4^+^ cells was then determined by FACS analysis. For Foxp3 expression, isolated cells were stained with Alexa Fluor 488 anti-mouse CD4 and PE anti-mouse CD25. Following fixation and permeabilization, cells were then stained with Alexa Flour 647 anti-mouse Foxp3 followed by FACS analysis to determine Foxp3 expression in CD4^+^CD25^+^ cells.

### Statistical analysis

Experimental results were given as mean ± SEM. For each measured variable, the Shapiro-Wilk normality test was performed to assess whether values were normally distributed. For normally distributed variables, comparisons between two groups were done by using the unpaired *t* test, whereas comparisons between multiple groups were done by one-way ANOVA followed by Bonferroni post hoc test. For variables displaying a non-Gaussian distribution, comparisons between two groups were done by using the Mann-Whitney *U* test. Statistical significance was determined as *p* values ≤ 0.05. All statistical analysis was performed by using the GraphPad Prism 8 software (La Jolla, CA).

## Results

### DMI ameliorates disease severity in chronic EAE

**Fig. 1:**
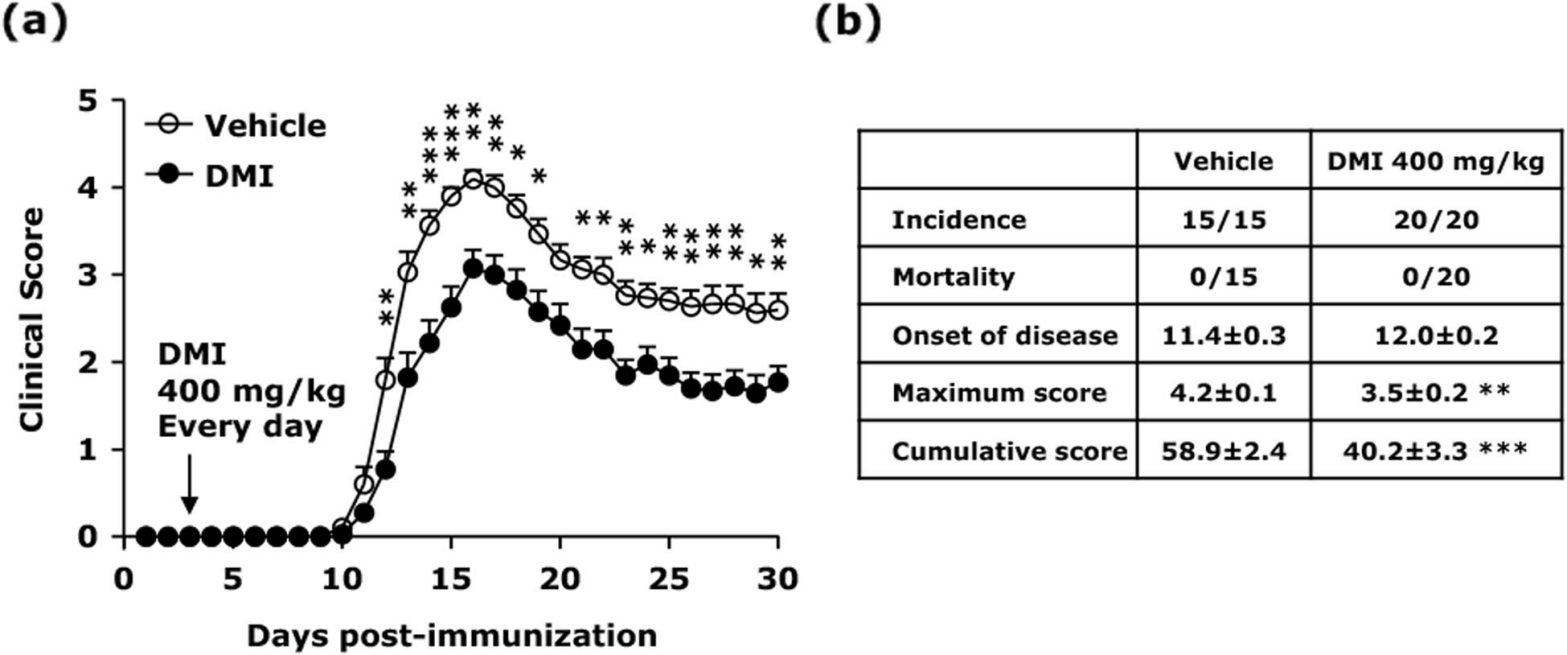
DMI ameliorates disease severity in chronic EAE. C57BL/6 mice were immunized with MOG_35–55_ and administered i.p. with vehicle (*n* = 15) or 400 mg/kg DMI (*n* = 20) every day starting from day 3 post-immunization. **a** The clinical score of EAE animals was followed for a period of 30 days. **b** The incidence and mortality rate of vehicle- and DMI-treated EAE mice were accessed, and the mean ± SEM of onset of disease, maximum score, and cumulative score (day 1 to day 30 post-immunization) in vehicle- and DMI-treated EAE was also calculated. Statistical significance was determined as *p < 0.05, **p < 0.01, and ***p < 0.001 by Mann-Whitney *U* test

To investigate the potential therapeutic effect of DMI in EAE, C57BL/6 mice were immunized with MOG_35–55_ followed by vehicle or DMI administration every day starting from day 3 post-immunization. The clinical score of vehicle- and DMI-treated EAE mice was assessed for a period of 30 days. Our results show that although DMI 300 mg/kg treatment did not offer a protection against EAE (Fig. S1A), DMI 400 mg/kg treatment exerted a protective effect and ameliorated disease severity in EAE (Fig. 1a). The maximum disease score in DMI-treated EAE mice was lower compared to that in vehicle-treated EAE mice (vehicle 4.2 ± 0.1 vs. DMI 3.5 ± 0.2; Fig. 1b). Further comparison of cumulative disease score between vehicle- and DMI-treated EAE mice revealed that DMI-treated EAE mice had a significant lower cumulative disease score than vehicle-treated EAE controls (vehicle 58.9 ± 2.4 vs. DMI 40.2 ± 3.3; Fig. 1b). Furthermore, EAE mice were treated with DMI 500 mg/kg or 600 mg/kg to assess whether increased doses of DMI would offer a better protection against EAE. The outcomes of disease onset and maximum disease score in EAE were comparable among DMI treatment doses of 400, 500, and 600 mg/kg (Fig. 1b and Fig. S1B). Although DMI treatment of 500 mg/kg and 600 mg/kg slightly reduced cumulative disease score compared to DMI treatment of 400 mg/kg in EAE, the differences did not reach statistical significance (data not shown). Thus, the DMI dose of 400 mg/kg was selected to continue with the studies. Taken altogether, these results demonstrate that DMI confers protection against EAE through ameliorating disease severity in EAE.

### DMI alleviates BBB disruption in EAE

**Fig. 2:**
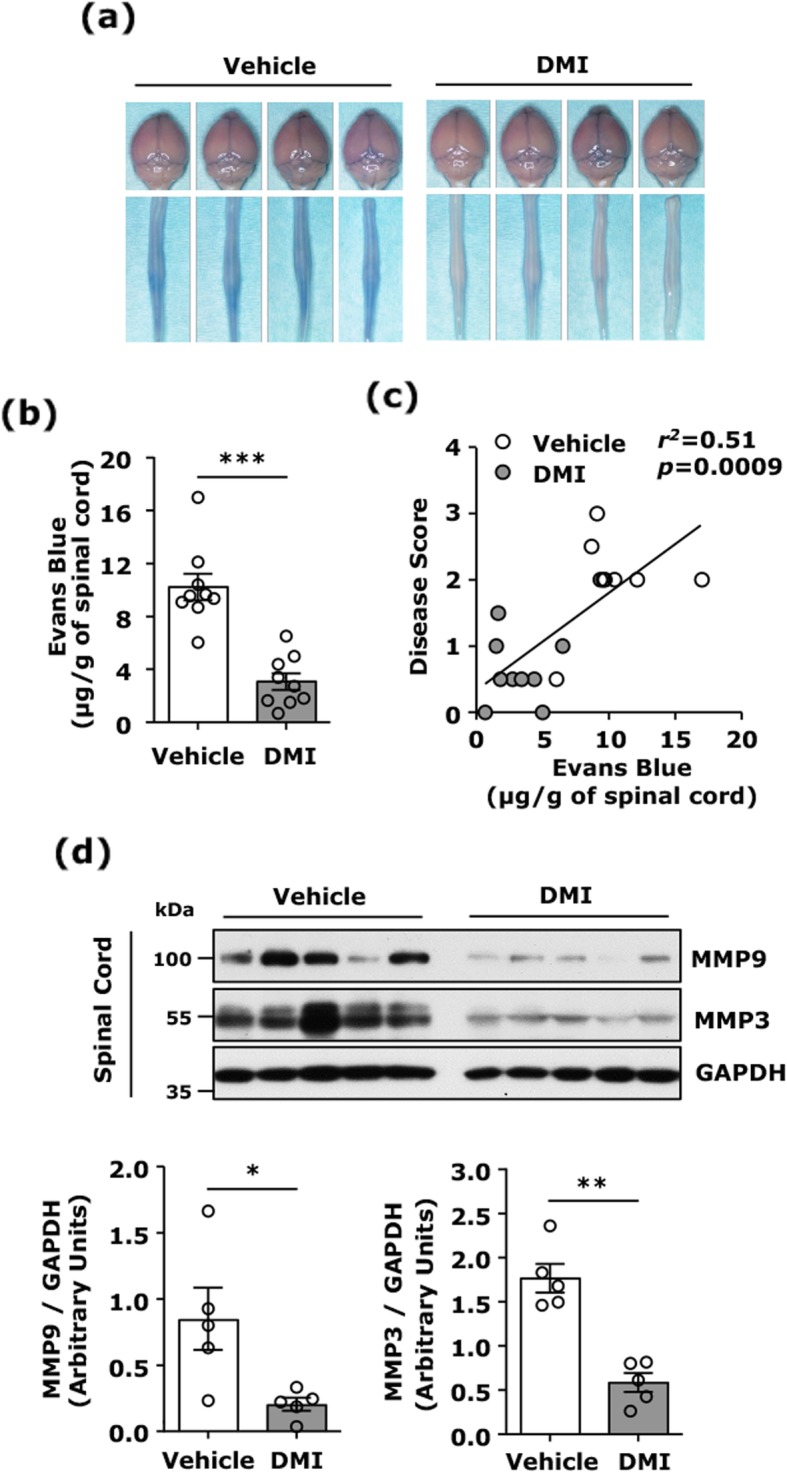
DMI alleviates BBB disruption. C57BL/6 EAE animals were administered i.p. with vehicle or DMI (400 mg/kg) every day starting from day 3 post-immunization. At day 12 post-immunization, vehicle- and DMI-treated EAE mice (*n* = 9/group) were subjected to i.v. administration of Evans blue. **a** EAE mice were sacrificed at 2 h post-Evans blue administration. The brain and spinal cord of vehicle- and DMI-treated EAE mice were harvested to assess the leakage of Evans blue. The four representative images of the brain and spinal cord harvested from vehicle- and DMI-treated EAE mice are shown. **b** The amount of Evans blue in the spinal cord tissues was measured and quantified. Statistical significance was determined as ***p < 0.001 by unpaired *t* test. **c** The correlation between Evans blue leakage and disease score was determined by linear regression analysis. **d** The spinal cord tissues harvested from vehicle- and DMI-treated EAE mice (*n* = 5/group) were homogenized and subjected to western blot analysis of MMP3 and MMP9 expression followed by quantification. Statistical significance was determined as *p < 0.05 and **p < 0.01 by Mann-Whitney *U* test

BBB disruption followed by inflammatory immune cell infiltration is a hallmark of immune-mediated inflammation of the CNS in MS and EAE [20, 21]. To elucidate whether DMI treatment alleviates BBB disruption in EAE, mice immunized with MOG_35–55_ and treated with vehicle or DMI were subject to Evans blue administration, and the CNS tissues were then harvested to assess BBB integrity. Our results show that there was an increased Evans blue leakage in the spinal cord of vehicle-treated EAE mice, suggesting BBB integrity was compromised. In contrast, Evans blue leakage was not clearly observed in the spinal cord of DMI-treated EAE mice (Fig. 2a). We then quantified the amount of Evans blue leakage in the spinal cord tissue of vehicle- and DMI-treated EAE mice, and our results show there was a significant reduction of Evans blue leakage in the spinal cord of DMI-treated EAE mice compared to that in vehicle-treated EAE mice (Fig. 2b). In addition, we observed that DMI-mediated mitigation of BBB disruption was highly correlated with DMI-mediated alleviation of disease severity in EAE mice (Fig. 2c).

Induction of MMP3 and MMP9 expression leads to BBB disruption in neuroinflammatory diseases, including MS/EAE [22–24]. To elucidate whether BBB disruption in the spinal cord of EAE mice is due to increased production of MMP3 and MMP9, the spinal cord tissues harvested from vehicle- and DMI-treated EAE mice were lysed and subjected to western blots analysis to determine MMP3 and MMP9 expression. We observed MMP3 and MMP9 were highly expressed in the spinal cord of vehicle-treated EAE mice. Conversely, MMP3 and MMP9 were strongly inhibited in the spinal cord of DMI-treated EAE mice (Fig. 2d). Altogether, these results suggest that DMI-mediated suppression of MMP3 and MMP9 production may contribute to the alleviation of BBB disruption in the spinal cord of DMI-treated EAE mice.

### DMI suppresses MG activation in EAE

**Fig. 3:**
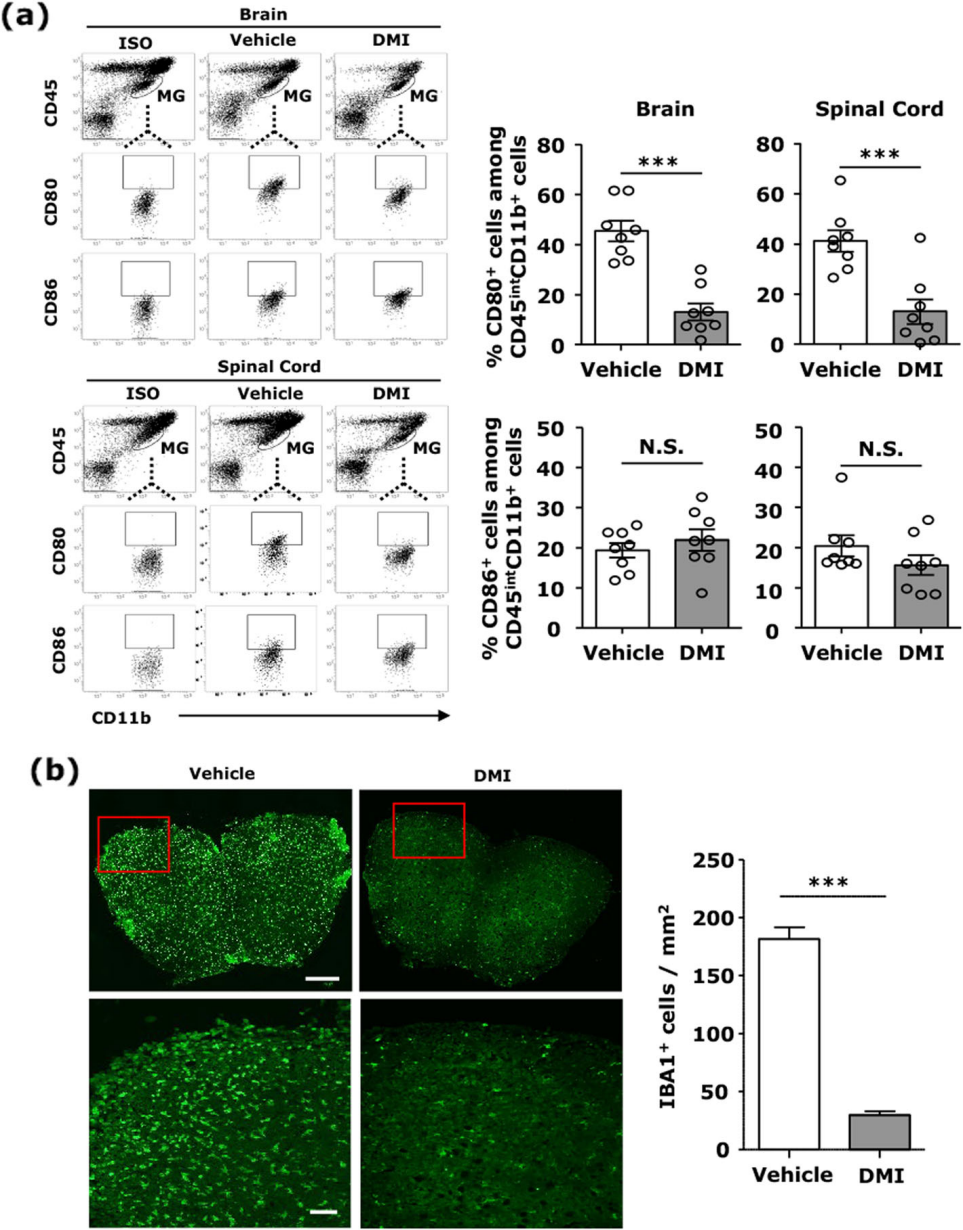
DMI suppresses MG activation in EAE. **a** C57BL/6 mice (*n* = 8/group) subjected to EAE were administered with vehicle or DMI (400 mg/kg) every day starting from day 3 post-immunization. At day 12–14 post-immunization, mononuclear cells were isolated from the brain and spinal cord of vehicle- and DMI-treated EAE mice. The isolated cells were then subjected to staining of CD45 and CD11b with CD80 or CD86 followed by FACS analysis. MG were determined based on their expression of CD45^int^CD11b^+^, and the surface expression of CD80 and CD86 on CD45^int^CD11b^+^ cells was then determined. Isotype controls (ISO) were used as a negative control to determine MG positive for CD80 or CD86 expression. **b** At day 12 post-immunization, the lumbar region of spinal cord tissues harvested from vehicle- and DMI-treated EAE mice (n = 5/group) was subjected to immunofluorescence analysis of IBA1 expression. The number of IBA1^+^ cells was calculated and quantified. Scale bars, top 200 μm and bottom 100 μm. Statistical significance was determined as ***p < 0.001 and N.S., no significant difference by unpaired *t* test

To investigate whether DMI exerts a modulatory effect on MG activation, the brain and spinal cord were harvested from vehicle- and DMI-treated EAE mice followed by mononuclear cell isolation. The isolated cells were subjected to FACS analysis to determine MG maturation status based on their expression of CD80 and CD86. Although there was no significant difference in the number of CD86^+^ MG, CD80^+^ MG were largely reduced in the brain and spinal cord of DMI-treated EAE mice compared to that in vehicle-treated EAE controls (Fig. 3a). In addition, IBA1^+^ MG/macrophages were also determined in the spinal cord, and our results show that the number of IBA1^+^ cells was significantly lower in the lumbar regions of spinal cord of DMI-treated EAE mice compared to that in vehicle-treated EAE controls. (Fig. 3b). Altogether, these results demonstrate that DMI suppresses MG activation in EAE.

### DMI inhibits MG activation and inflammatory cytokine production and enhances Nrf2/HO-1 defense pathway in MG

**Fig. 4:**
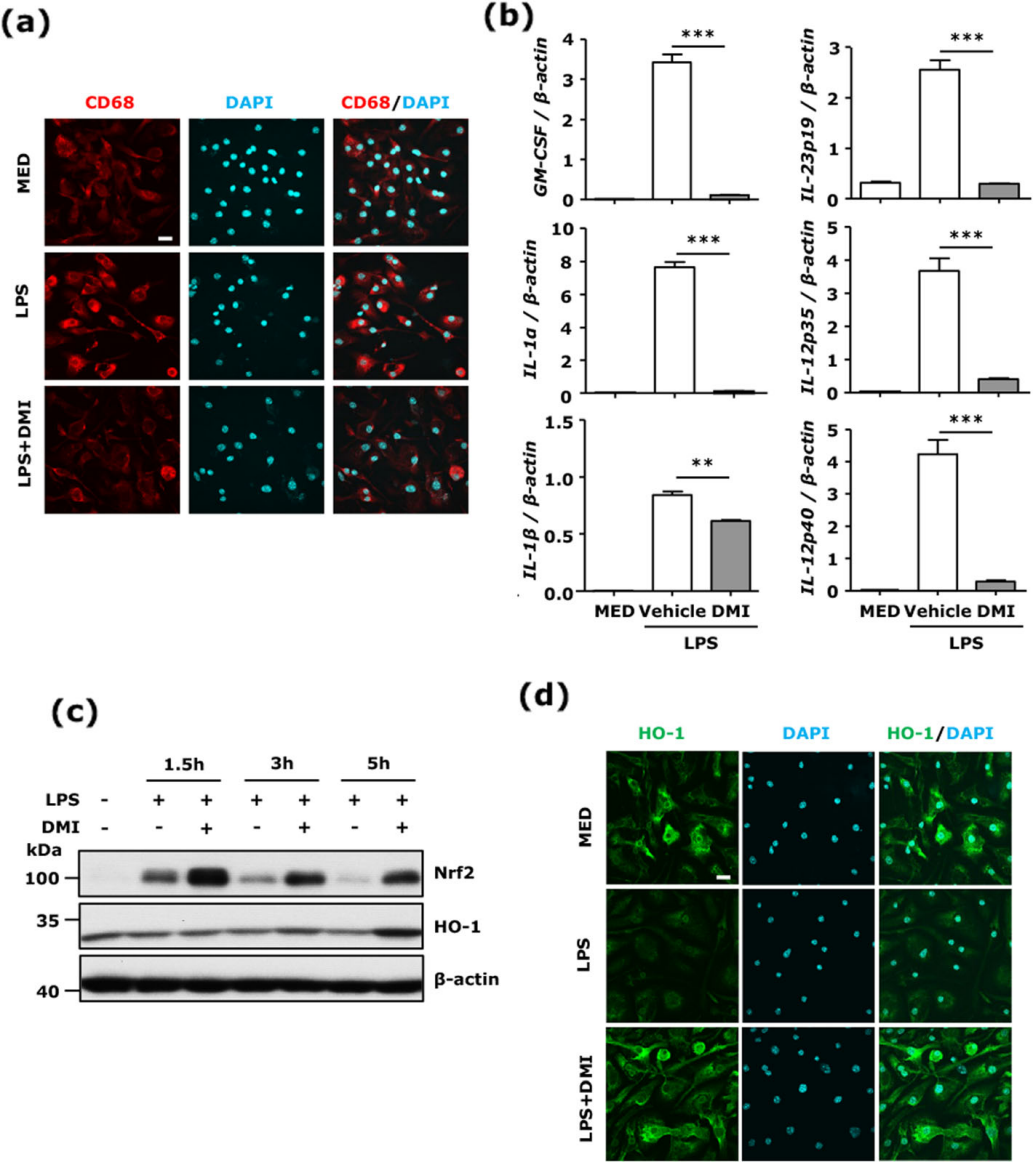
DMI inhibits MG activation and inflammatory cytokine production and enhances Nrf2/HO-1 defense pathway in MG. Primary MG or BV2 cells were left untreated (MED) or pretreated with vehicle or DMI 150 μM for 1 h followed by LPS (100 ng/ml) stimulation. **a** 24 h after LPS stimulation, primary MG were subjected to CD68 staining to assess MG activation. The representative confocal images of three independent experiments are shown. Scale bar, 20 μm. **b** 1.5 h after LPS stimulation, primary MG were collected and subjected to RNA extraction followed by Q-PCR analysis for mRNA expression of GM-CSF, IL-23p19, IL-12p35, IL-12p40, IL-1α, and IL-1β. The representative results of three independent experiments are shown. Statistical significance was determined as **p < 0.01 and ***p < 0.001 by one-way ANOVA with post-hoc Bonferroni’s multiple comparison test. **c** 1.5, 3, and 5 h after LPS stimulation, BV2 cells were collected and subjected to western blot analysis for Nrf2 and HO-1 expression. The representative results of three independent experiments are shown. **d** 8 h after LPS stimulation, primary MG were subjected to immunofluorescence analysis to determine HO-1 expression. The representative confocal images of four independent experiments are shown. Scale bar, 20 μm

To further confirm the inhibitory effect of DMI on MG activation, primary MG were cultured and activated with LPS in the presence or absence of DMI. MG were then subjected to immunofluorescence staining and Q-PCR analysis to determine the activation marker CD68 expression and inflammatory cytokine production, respectively. LPS strongly induced CD68 expression in primary MG. However, LPS-induced CD68 expression was abolished in the presence of DMI (Fig. 4a). Furthermore, GM-CSF, which has been shown to promote neuroinflammation and mediate immunopathology in EAE [25, 26], was highly upregulated by LPS in MG. However, LPS-induced GM-CSF upregulation was abolished by DMI in MG. Moreover, inflammatory cytokines IL-23p19 required for Th17 differentiation and IL-12p35 and IL-12p40 required for Th1 differentiation were upregulated in LPS-treated MG, but were strongly suppressed in LPS + DMI-treated MG. DMI also suppressed LPS-induced IL-1α and IL-1β expression in MG (Fig. 4b).

Induction of Nrf2/HO-1 pathway has been shown to promote anti-oxidant and anti-inflammatory effects [27, 28]. It has been shown that DMI induced Nrf2 activation in macrophages [10]. Here, we investigated whether DMI induces Nrf2/HO-1 pathway in MG. Microglial cell lines, BV2 cells, were treated with LPS in the presence or absence of DMI for a time course. Our results show that DMI enhanced Nrf2 expression at early time points and upregulated HO-1 expression at late time points in BV2 cells (Fig. 4c). DMI-mediated HO-1 upregulation was further confirmed in primary MG by immunofluorescence staining, and our results show that HO-1 expression was strongly induced by DMI in LPS-treated MG (Fig. 4d).

### DMI represses the CNS infiltration of encephalitogenic Th1 and Th17 cells in EAE

**Fig. 5:**
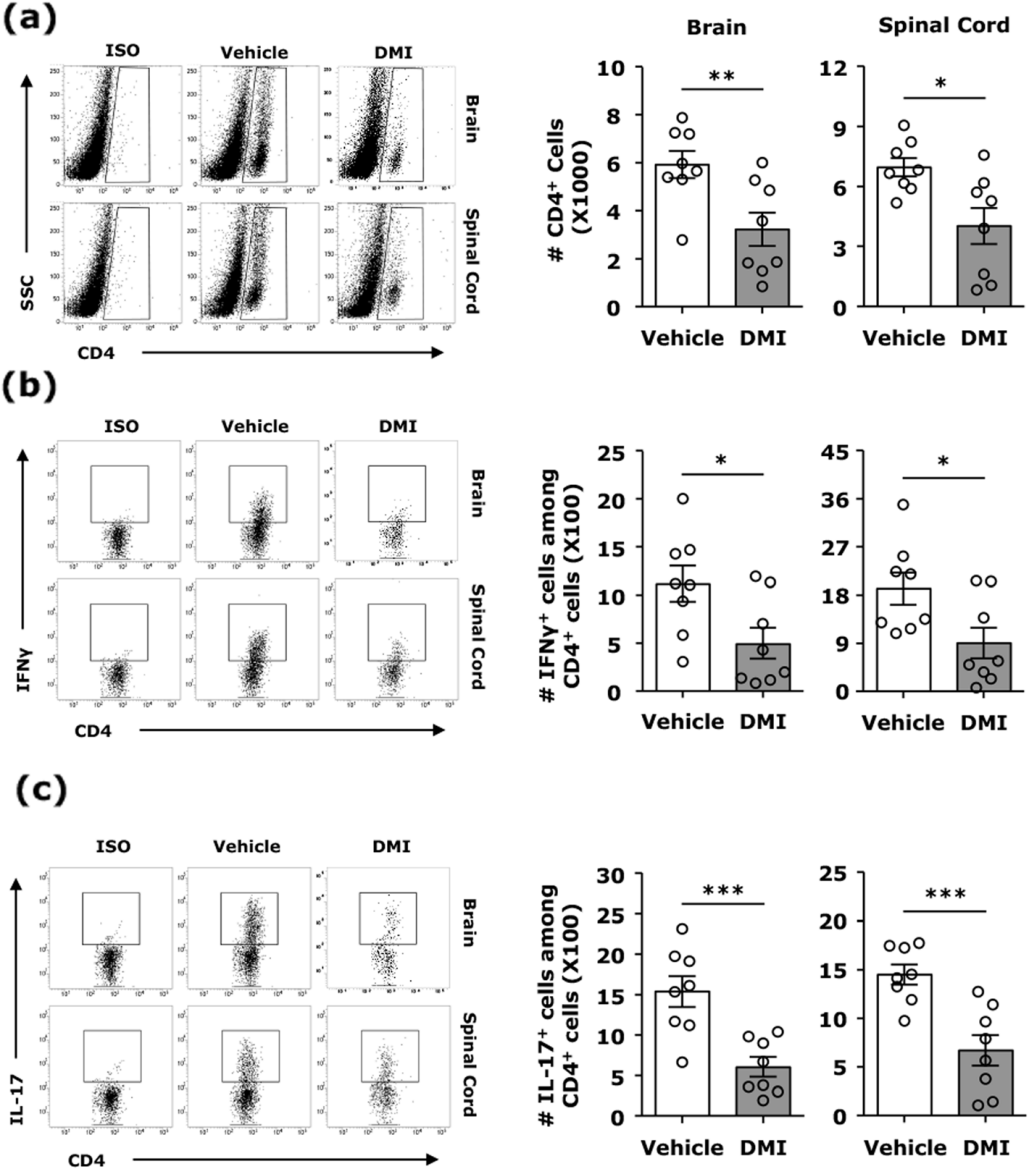
DMI represses the CNS infiltration of encephalitogenic Th1 and Th17 cells in EAE. C57BL/6 mice (*n* = 8/group) were subjected to EAE induction followed by i.p. administration with vehicle or DMI (400 mg/kg) every day starting from day 3 post-immunization. At day 12–14 post-immunization, mononuclear cells were isolated from the brain and spinal cord of vehicle- and DMI-treated EAE mice. The number of (**a**) CD4^+^ T cells, (**b**) IFNγ-expressing CD4^+^ T cells, and (**c**) IL-17-expressing CD4^+^ T cells in the brain and spinal cord of vehicle- and DMI-treated EAE mice was determined by FACS analysis. ISO were used as a negative control to determine cells positive for the surface expression of CD4 or CD4^+^ cells positive for the intracellular expression of IFNγ or IL-17. Statistical significance was determined as *p < 0.05, **p < 0.01, and ***p < 0.001 by unpaired *t* test

Given that the CNS infiltration of encephalitogenic Th1 and Th17 cells plays a pivotal role in the pathogenesis of EAE, we tested whether DMI suppresses pathogenic CD4^+^ T cell infiltration of the CNS. Mononuclear cells were isolated from the brain and spinal cord of vehicle- and DMI-treated EAE mice, and the isolated cells were then subjected to FACS analysis to determine the number of the CNS infiltrating CD4^+^ T cells as well as IFNγ- and IL-17-expressing CD4^+^ T cells. Significant CD4^+^ T cell infiltrates were observed in the brain and spinal cord of vehicle-treated EAE mice. In contrast, CD4^+^ T cell infiltrates were suppressed in the brain and spinal cord of DMI-treated EAE mice (Fig. 5a). Further analysis of Th1 and Th17 infiltration of the CNS revealed that both Th1 and Th17 infiltrates were decreased in the brain and spinal cord of DMI-treated EAE mice compared to those in vehicle-treated EAE controls (Fig. 5b, c). In addition, the frequency of T regulatory cells (Tregs) in the CNS of vehicle- and DMI-treated EAE mice was evaluated. Our results show that the frequency of Foxp3-expressing CD4^+^CD25^+^ cells in the brain and spinal cord of DMI-treated EAE mice was comparable to that in vehicle-treated EAE controls (Fig. S2), suggesting that DMI did not alter the frequency of Tregs in the CNS of EAE. Altogether, these results demonstrate that DMI represses encephalitogenic Th1 and Th17 cell infiltration of the CNS that may contribute to ameliorated disease severity in DMI-treated EAE mice.

### DMI suppresses Th1 and Th17 differentiation in vivo and in vitro

**Fig. 6:**
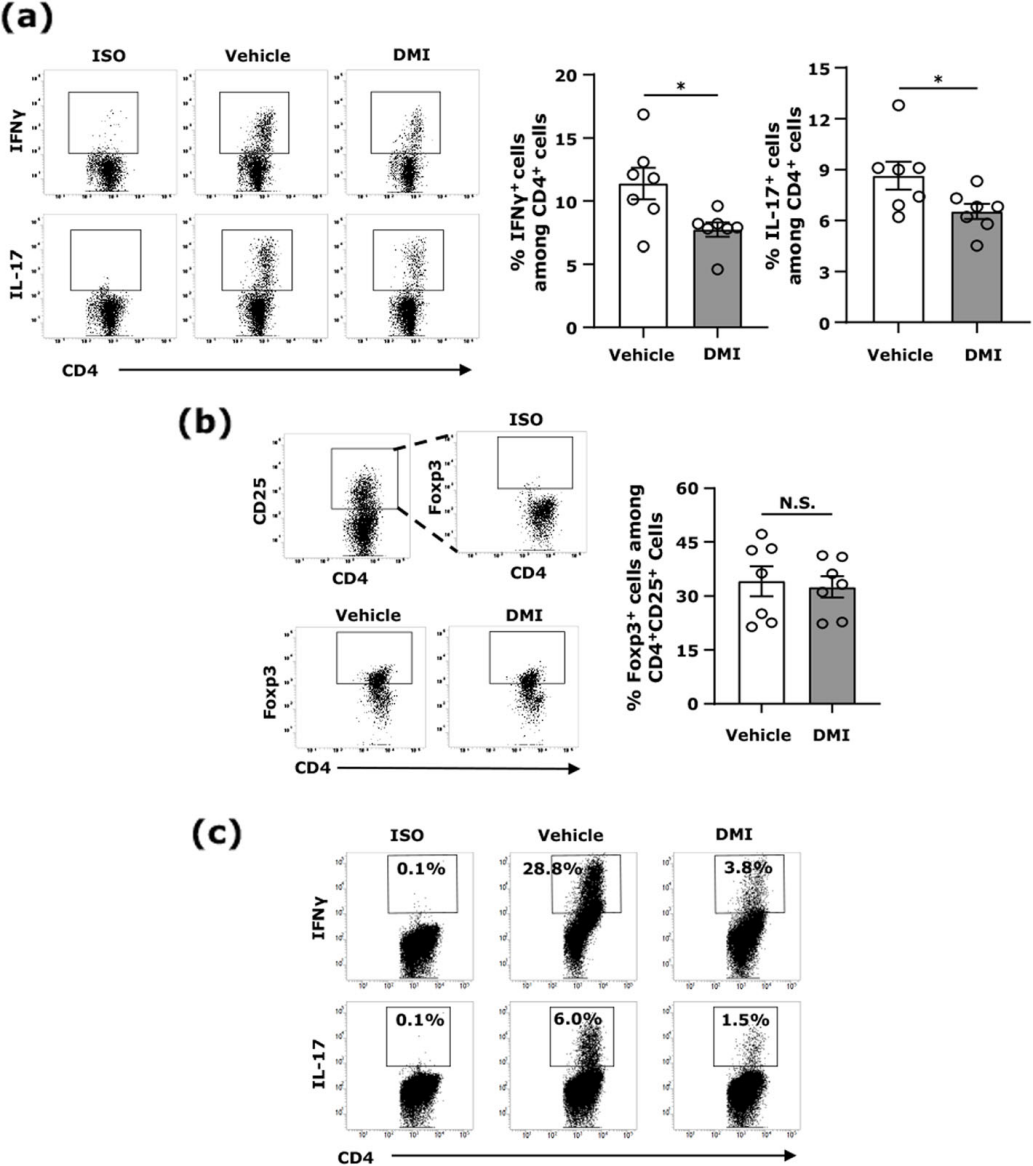
DMI suppresses Th1 and Th17 differentiation in vivo and in vitro*.* Cells were harvested from the spleen of vehicle- and DMI (400 mg/kg)-treated EAE mice (*n* = 7/group) at day 10 post-immunization. **a** IFNγ- and IL-17-expressing CD4^+^ cells and **b** Foxp3-expressing CD4^+^CD25^+^ cells were determined by FACS analysis. ISO were used as a negative control to determine CD4^+^ cells positive for the intracellular expression of IFNγ or IL-17 or CD4^+^CD25^+^ cells positive for the nuclear expression of Foxp3. Statistical significance was determined as *p < 0.05. N.S., no significant difference by unpaired *t* test. **c** Naive splenocytes were harvested and polarized into Th1 or Th17 condition in the absence or presence of DMI (150 μM). Forty-eight hours later, cells were harvested and subjected to FACS analysis for the intracellular expression of IFNγ or IL-17 in CD4^+^ T cells. ISO were used as a negative control to determine CD4^+^ cells positive for the intracellular expression of IFNγ or IL-17. The representative data of three independent experiments are shown

To elucidate whether DMI modulates peripheral Th1 and Th17 differentiation in EAE, cells were isolated from the spleen and cervical lymph nodes (CLNs) of vehicle- and DMI-treated EAE mice at day 10 post-immunization followed by FACS analysis for the intracellular expression of IFNγ and IL-17 in CD4^+^ cells. Although a similar frequency of IFNγ- and IL-17-expressing CD4^+^ T cells was observed in the CLNs of vehicle- and DMI-treated EAE mice (Fig. S3A), the frequency of IFNγ- and IL-17-expressing CD4^+^ T cells was decreased in the spleen of DMI-treated EAE mice compared to that in vehicle-treated EAE controls (Fig. 6a). In addition, to evaluate whether DMI treatment affects Treg differentiation in the periphery of EAE mice, cells isolated from the spleen and CLNs of vehicle- and DMI-treated EAE mice were subjected to FACS analysis to determine the frequency of Tregs. Our results show that DMI treatment did not alter the frequency of Foxp3-expressing CD4^+^CD25^+^ cells in the spleen and CLNs compared to vehicle treatment in EAE (Fig. 6b and Fig. S3B). Finally, to determine whether DMI exerts a direct suppressive effect on the differentiation of pathogenic T cells, naive splenocytes were polarized into Th1 or Th17 cells in the presence or absence of DMI followed by FACS analysis for the intracellular expression of IFNγ and IL-17 in CD4^+^ T cells. We observed that DMI suppressed the frequency of IFNγ- and IL-17-expressing CD4^+^ cells in the polarized Th1 and Th17 splenocyte cultures, respectively, suggesting that DMI possesses a direct inhibitory effect on Th1 and Th17 differentiation (Fig. 6c). Taken altogether, our results demonstrate that DMI suppresses the differentiation of pathogenic Th1 and Th17 cells in the periphery that may contribute to the decreased infiltration of encephalitogenic Th1 and Th17 cells in the CNS of DMI-treated EAE mice.

### DMI alleviates the severity of relapse in relapsing-remitting EAE

**Fig. 7:**
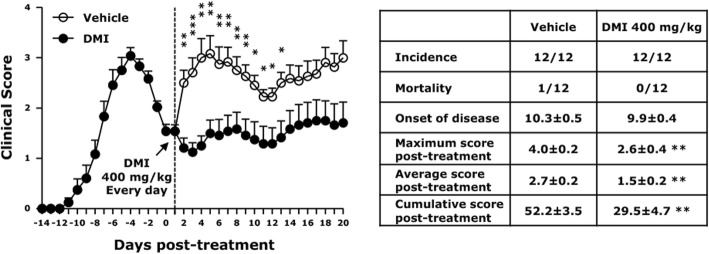
DMI alleviates the severity of relapse in relapsing-remitting EAE. SJL/J mice (*n* = 12/group) were immunized with PLP_139–151_ to induce EAE. Following the first remission and before the onset of relapse, EAE mice were administered i.p. with vehicle or DMI (400 mg/kg) every day. The clinical score of EAE mice was evaluated for a period of 20 days. The incidence and mortality rate of vehicle- and DMI-treated EAE mice were accessed, and the mean ± SEM of onset of disease, maximum score, average score, and cumulative score of day 1 to day 20 post-treatment in vehicle- and DMI-treated EAE was calculated. Statistical significance was determined as *p < 0.05, **p < 0.01, and ***p < 0.001 by Mann-Whitney *U* test

To further explore whether DMI possesses a therapeutic effect on alleviating the severity of relapse in EAE, relapsing-remitting EAE was induced in SJL/J mice, and EAE mice were administered with vehicle or DMI starting from the first remission before the onset of relapse. EAE mice treated with vehicle developed disease relapse following remission with the maximum disease score and cumulative disease score of post-treatment reaching 4.0 ± 0.2 and 52.2 ± 3.5, respectively. In contrast, EAE mice treated with DMI displayed alleviated severity of relapse with the maximum disease score and cumulative disease score of post-treatment only reaching 2.6 ± 0.4 and 29.5 ± 4.7, respectively (Fig. 7). Collectively, these results demonstrate that DMI possesses a therapeutic effect on alleviating the severity of relapse in EAE, and suggest that DMI can be developed as a novel therapy for the treatment of MS/EAE.

## Discussion

Itaconate, a metabolite synthesized by the enzyme encoded by IRG1, has recently emerged as a regulator of macrophage functions [10–12, 29]. It has been shown that itaconate exerts an anti-inflammatory effect through suppressing succinate dehydrogenase (SDH) activity, a crucial pro-inflammatory regulator [30]. In addition, itaconate was reported to activate Nrf2/HO-1 pathway which possesses anti-oxidant and anti-inflammatory effects [10, 31]. Moreover, itaconate has been shown to inhibit LPS-induced IκBς activation in macrophages and IL-17-induced IκBς activation in keratinocytes, leading to suppression of IL-6 and IL-12 production and amelioration of psoriatic pathology, respectively [12]. Presently, the anti-inflammatory effect of itaconate was mostly reported in macrophages and peripheral inflammatory immune responses, and little is known with regard to its effect on MG and the CNS inflammation. Thus, in this study, we aimed to explore the effect of itaconate on MG and neuroinflammation using EAE models. Similar to its immunomodulatory effect on macrophages, DMI suppressed MG activation, and this immunomodulatory effect of DMI on MG was observed both in vitro and in vivo. In vitro, DMI inhibited LPS-induced inflammatory cytokine production in MG and suppressed LPS-induced MG activation. In vivo, DMI suppressed MG activation in EAE in which the number of CD80^+^ MG and IBA1^+^ MG/macrophages in the CNS of DMI-treated EAE mice was significantly lower than that in vehicle-treated EAE controls. Thus, our findings demonstrate the immunomodulatory effect of DMI on LPS-induced MG activation and MG-mediated neuroinflammation in EAE.

Although the molecular mechanisms by which DMI suppresses MG activation and neuroinflammation in EAE were not investigated in this study, we speculated that the protective effect of DMI in EAE may be mediated through two major mechanisms, namely activation of Nrf2/HO-1 pathway and suppression of SDH/HIF-1α activity. The induction of Nrf2/HO-1 pathway has been shown to confer a protection against EAE [19, 32–34]. Similar to the previous findings of DMI induced Nrf2 activation in macrophages [10, 12], we observed DMI enhanced Nrf2 and HO-1 expression in MG. Furthermore, itaconate has been shown to inhibit SDH activity that leads to the repression of succinate accumulation and subsequent HIF-1α activation as well as IL-1β production [29, 30, 35, 36]. As HIF-1α activation was reported to promote Th17 differentiation [37, 38], itaconate-mediated repression of HIF-1α activation may result in hindering Th17 differentiation. Taken altogether, it suggests that Nf2/HO-1 pathway activation and SDH/HIF-1α activity suppression may account for the two major molecular mechanisms underlying the protective effect of DMI in EAE, although it would require further studies to demonstrate the proposed mechanisms.

The BBB is a complex organization composed of cerebral endothelial cells, pericytes, and basal lamina, surrounded by astrocytes and perivascular macrophages [39]. These cells separate and form the compartments of the cerebral vascular space during homeostasis. The BBB dysregulation and the CNS transendothelial migration of activated immune cells are associated with cerebrovascular abnormalities observed in MS/EAE. Similar to the previous findings [40, 41], we observed profound extravasation of Evans blue in the spinal cord of EAE mice. However, Evans blue extravasation was significantly reduced in the spinal cord of DMI-treated EAE mice, suggesting that DMI possesses a protective effect on the alleviation of BBB disruption in EAE. Notably, DMI-mediated alleviation of BBB disruption is correlated with reduced Th1 and Th17 infiltration into the spinal cord of DMI-treated EAE mice. Studies have shown that MMP3 and MMP9 play a detrimental role in BBB disruption [20, 21]. Consistently, our results show that MMP3 and MMP9 were highly upregulated in the spinal cord of EAE mice. Most importantly, EAE-induced MMP3 and MMP9 expression in the spinal cord were strongly suppressed by DMI treatment that might contribute to the alleviation of BBB disruption in the spinal cord of DMI-treated EAE mice.

Currently, more than ten disease-modifying therapies are available for relapsing forms of MS. Among them, interferon beta, a cytokine with immunomodulatory properties, has been shown to modify disease through inhibiting Th1 and Th17 differentiation, and promoting IL-10 production [42–46]. Glatiramer acetate, a random polymer composed of four amino acids found in myelin basic protein, was reported to shift T cell population from Th1 cells to Th2 cells, resulting in the suppression of the inflammatory response [47, 48]. Natalizumab, a humanized monoclonal antibody targeting the α4 subunit of VLA4 adhesion molecule on the surface of lymphocyte, has been shown to prevent the binding between VLA4 and VCAM-1 on brain vascular endothelium, resulting in suppressed pathogenic T cell infiltration of the CNS [49]. Dimethyl fumarate (DMF), an orally administered fumarate ester, was recently approved by FDA. Although the precise mechanism of action of DMF in MS is not completely characterized, it was reported that DMF is rapidly hydrolyzed to its active metabolite, monomethyl fumarate (MMF) by esterases, and MMF exerts neuroprotective effects by activating Nrf2/HO-1 pathway [50]. In addition, we have previously shown that DMF attenuated LPS-induced MG activation and long-term memory deficits through Nrf2-dependent and independent mechanisms [51]. In this study, we identified that DMI induced Nrf2/HO-1 pathway in primary MG and suppressed MG activation in EAE, suggesting that DMI may exert a similar protective mechanism as DMF.

Although we observed DMI ameliorated disease severity in EAE, further studies would be required to address the limitations on the current study. For instance, as we show that DMI inhibited Th1/Th17 differentiation in vivo and in vitro, it would be of interest to investigate whether DMI-induced Nrf2/HO-1 activation has a direct effect on the suppression of pathogenic Th1/Th17 differentiation. In addition, as we found that DMI suppressed MMP3 and MMP9 production that subsequently led to alleviated BBB disruption in the spinal cord of EAE, it would be important to elucidate the molecular mechanisms underlying the inhibitory effect of DMI on MMP3 and MMP9 production. Finally, as the CNS resident cells, including neurons, MG, astrocytes, and brain endothelial cells, and the CNS infiltrating immune cells may contribute to the production of MMP3 and MMP9 in the spinal cord of EAE, further studies would be required to dissect what cell types of MMP3 and MMP9 producers modulated by DMI treatment in EAE.

## Conclusions

In this study, we reported for the first time that DMI, an itaconate derivative, exerted a protective effect in chronic C57BL/6 EAE. We identified that DMI suppressed LPS-induced cell activation and inflammatory cytokine production in primary MG and ameliorated MG-mediated neuroinflammation in EAE. Furthermore, DMI alleviated EAE-induced BBB disruption, and this protective effect might be mediated through its inhibitory effect on MMP3 and MMP9 production. Moreover, DMI inhibited peripheral Th1/Th17 differentiation and repressed Th1/Th17 infiltration of the CNS in EAE. Finally, DMI exhibited a therapeutic effect on alleviating the severity of relapse in relapsing-remitting SJL/J EAE. In conclusion, we demonstrate that DMI suppresses neuroinflammation and ameliorates disease severity in EAE through multiple cellular and molecular mechanisms, suggesting that DMI can be developed as a novel therapeutic agent for the treatment of MS/EAE through its immunomodulatory and anti-inflammatory properties.

## Supplementary information

**Additional file 1: Figure S1.** C57BL/6 mice were immunized with MOG_35-55_ and administered i.p. with vehicle or DMI every day starting from day 3 post-immunization. The clinical score of EAE mice treated with (A) vehicle, DMI 300 mg/kg (n=5/group), (B) DMI 500 mg/kg (n=10/group) or DMI 600 mg/kg (n=10/group) was followed for a period of 30 days. The incidence and mortality rate of vehicle- and DMI-treated EAE mice were accessed, and the mean ± SEM of onset of disease, maximum score and cumulative score (day 1 to day 30 post-immunization) in vehicle- and DMI-treated EAE was also calculated. Statistical significance was determined as: *p<0.05, **p<0.01 and ***p<0.001 by Mann-Whitney *U* test. **Figure S2.** C57BL/6 mice were immunized with MOG_35-55_ and administered i.p. with vehicle or 400 mg/kg DMI (n=8/group) every day starting from day 3 post-immunization. At day 12 post-immunization, animals were sacrificed, and the brains and spinal cords were harvested followed by mononuclear cell isolation. The isolated cells were then subjected to staining with anti-CD4 and anti-CD25 antibodies. After wash, cells were fixed, permeabilized and stained with anti-Foxp3 antibody followed by FACS analysis. CD4^+^ cells (3000-5000 events) were acquired from each brain and spinal cord sample, and the nuclear expression of Foxp3 in CD4^+^CD25^+^ cells was determined. Isotype controls (ISO) were used as a negative control to determine CD4^+^CD25^+^ cells positive for the nuclear expression of Foxp3. Data represent mean ± SEM. Statistical significance was determined as: N.S., no significant difference by unpaired *t* test. **Figure S3.** C57BL/6 mice were immunized with MOG_35-55_ and administered i.p. with vehicle or 400 mg/kg DMI (n=7/group) every day starting from day 3 post-immunization. At day 10 post-immunization, animals were sacrificed, and the superficial and deep cervical lymph nodes were harvested followed by cell isolation. Cells were then subjected to FACS analysis to determine (A) the intracellular expression of IFNγ and IL-17 in CD4^+^ cells or (B) the nuclear expression of Foxp3 in CD4^+^CD25^+^ cells. ISO were used as a negative control to determine CD4^+^ cells positive for the intracellular expression of IFNγ or IL-17 or CD4^+^CD25^+^ cells positive for the nuclear expression of Foxp3. Data represent mean ± SEM. Statistical significance was determined as: N.S., no significant difference by unpaired *t* test.

## Data Availability

The datasets of the current study are available from the corresponding author on a reasonable request.

## Abbreviations

MS: Multiple sclerosis
MG: Microglia
IRG1: Immunoresponsive gene 1
DMI: Dimethyl itaconate
EAE: Experimental autoimmune encephalomyelitis
BBB: Blood-brain barrier
PMA: Phorbol myristate acetate
RT: Room temperature
SDH: Succinate dehydrogenase
CLNs: Cervical lymph nodes
