# Supplementary material for: Dimethyl itaconate, an itaconate derivative, exhibits immunomodulatory effects on neuroinflammation in experimental autoimmune encephalomyelitis
Source: J Neuroinflammation. 2020 Apr 29;17:138. doi: 10.1186/s12974-020-01768-7 (PMC7191722; doi:10.1186/s12974-020-01768-7)
Supplement: Supplementary file 1 — Additional file 1: Figure S1. C57BL/6 mice were immunized with MOG35-55 and administered i.p. with vehicle or DMI every day starting from day 3 post-immunization. The clinical score of EAE mice treated with (A) vehicle, DMI 300 mg/kg (n=5/group), (B) DMI 500 mg/kg (n=10/group) or DMI 600 mg/kg (n=10/group) was followed for a period of 30 days. The incidence and mortality rate of vehicle- and DMI-treated EAE mice were accessed, and the mean ± SEM of onset of disease, maximum score and cumulative score (day 1 to day 30 post-immunization) in vehicle- and DMI-treated EAE was also calculated. Statistical significance was determined as: *p<0.05, **p<0.01 and ***p<0.001 by Mann-Whitney U test. Figure S2. C57BL/6 mice were immunized with MOG35-55 and administered i.p. with vehicle or 400 mg/kg DMI (n=8/group) every day starting from day 3 post-immunization. At day 12 post-immunization, animals were sacrificed, and the brains and spinal cords were harvested followed by mononuclear cell isolation. The isolated cells were then subjected to staining with anti-CD4 and anti-CD25 antibodies. After wash, cells were fixed, permeabilized and stained with anti-Foxp3 antibody followed by FACS analysis. CD4+ cells (3000-5000 events) were acquired from each brain and spinal cord sample, and the nuclear expression of Foxp3 in CD4+CD25+ cells was determined. Isotype controls (ISO) were used as a negative control to determine CD4+CD25+ cells positive for the nuclear expression of Foxp3. Data represent mean ± SEM. Statistical significance was determined as: N.S., no significant difference by unpaired t test. Figure S3. C57BL/6 mice were immunized with MOG35-55 and administered i.p. with vehicle or 400 mg/kg DMI (n=7/group) every day starting from day 3 post-immunization. At day 10 post-immunization, animals were sacrificed, and the superficial and deep cervical lymph nodes were harvested followed by cell isolation. Cells were then subjected to FACS analysis to determine (A) the intracellular [file 12974_2020_1768_MOESM1_ESM.pdf]

Figure S1

(A)

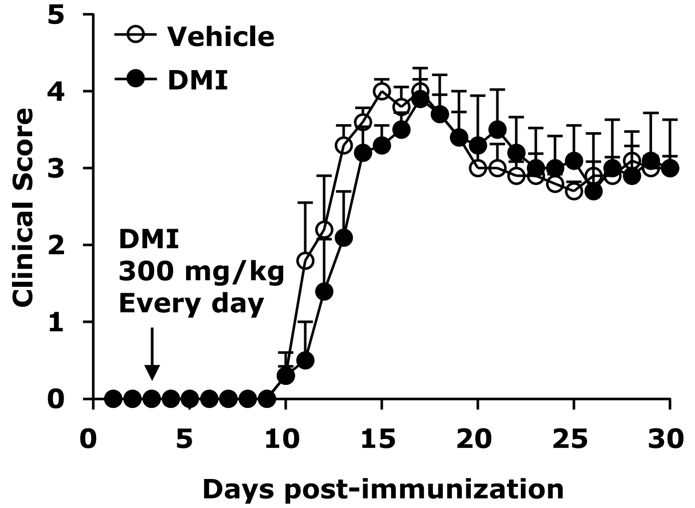

	Vehicle	DMI 300 mg/kg
Incidence	5/5	5/5
Mortality	0/5	0/5
Onset of disease	10.8±0.5	12.0±0.6
Maximum score	4.2±0.2	4.1±0.3
Cumulative score	62.3±3.5	59.1±7.5

(B)

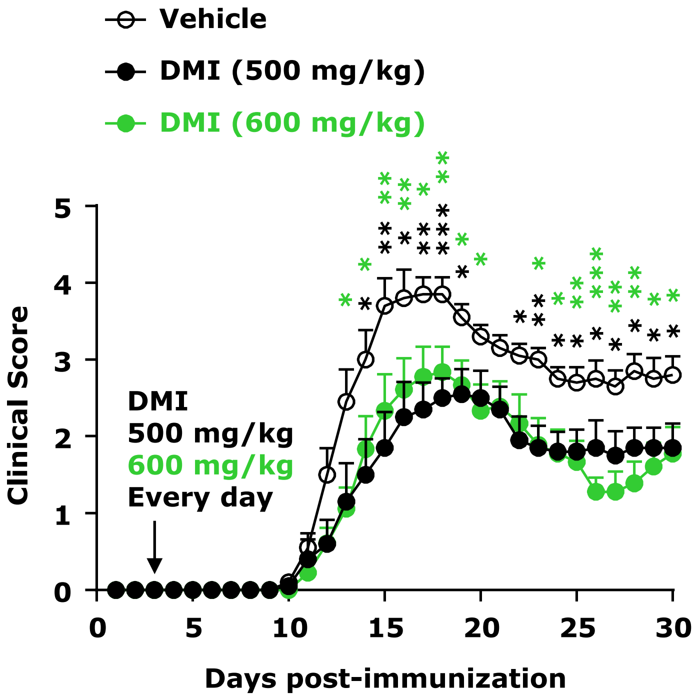

	Vehicle	DMI 500 mg/kg	DMI 600 mg/kg
Incidence	10/10	10/10	10/10
Mortality	0/10	0/10	1/10
Onset of disease	11.5±0.5	13.0±0.6	12.4±0.6
Maximum score	4.3±0.1	3.3±0.3 **	3.2±0.3 **
Cumulative score	58.1±3.3	36.6±5.0 *	36.5±4.6 ***

Figure S2

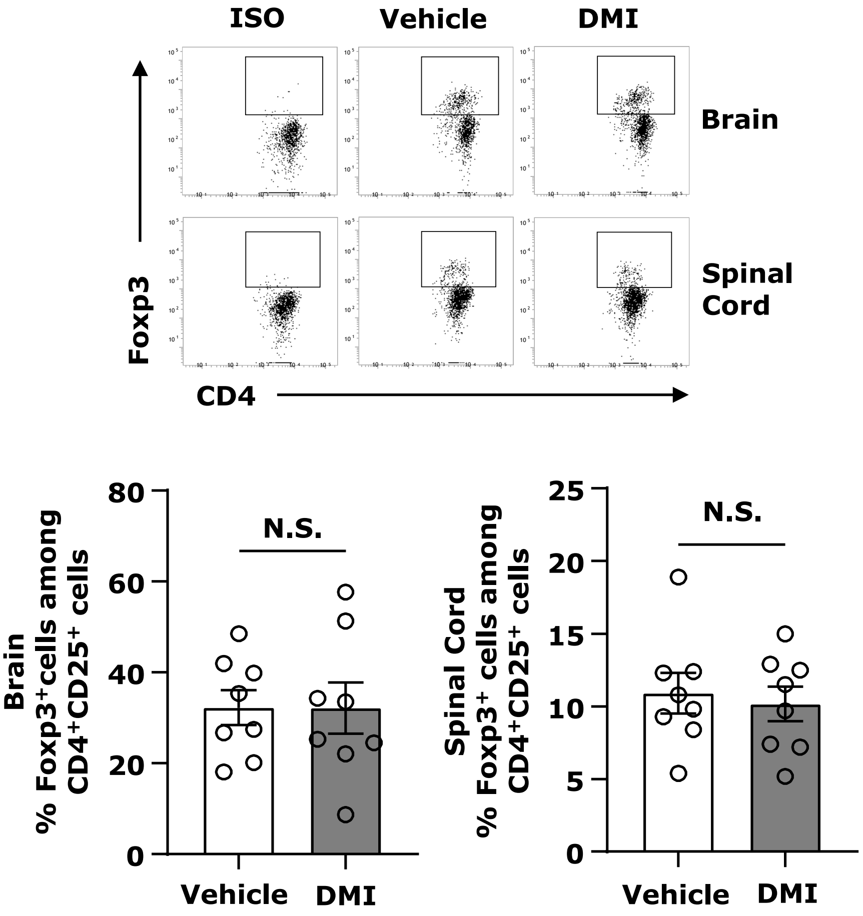
ISO
Vehicle
DMI
Brain
Spinal Cord
Foxp3
CD4
N.S.
Brain % Foxp3+cells among CD4+CD25+ cells
0
20
40
60
80
Vehicle
DMI
N.S.
Spinal Cord % Foxp3+ cells among CD4+CD25+ cells
0
5
10
15
20
25
Vehicle
DMI

Figure S3

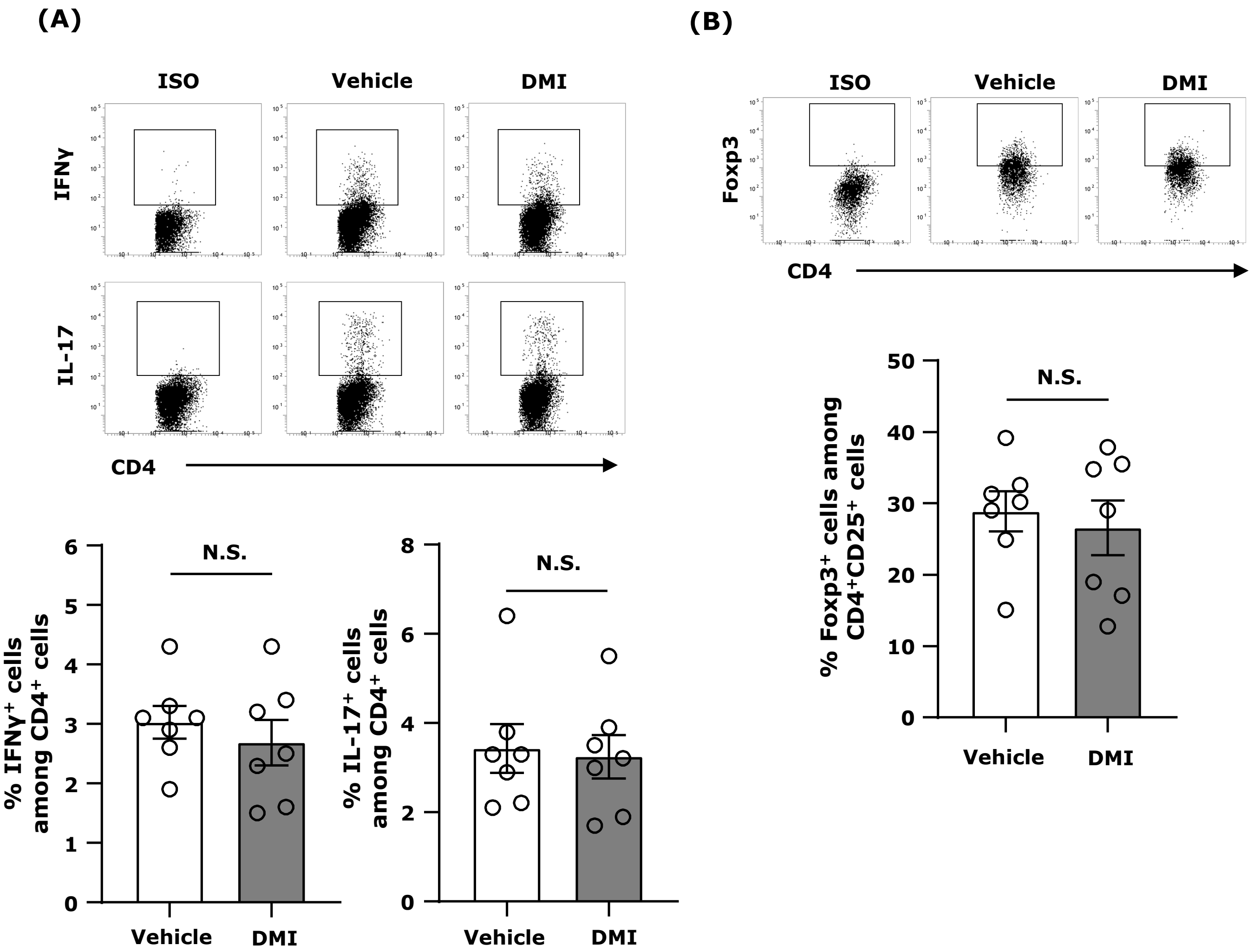

(A)
ISO
Vehicle
DMI
IFNγ
IL-17
CD4
% IFNγ+ cells among CD4+ cells
N.S.
Vehicle
DMI
% IL-17+ cells among CD4+ cells
N.S.
Vehicle
DMI
(B)
ISO
Vehicle
DMI
Foxp3
CD4
% Foxp3+ cells among CD4+CD25+ cells
N.S.
Vehicle
DMI
